# Hyperautofluorescence in Outer Retinal Layers Thinning

**DOI:** 10.1155/2014/741538

**Published:** 2014-09-09

**Authors:** Marina Bertolotto, Luigi Borgia, Michele Iester

**Affiliations:** ^1^Is.PreOftalmica, Via Antiochia 29r, 16129 Genoa, Italy; ^2^Anatomical-Clinical Laboratory for Functional Diagnosis and Treatment of Glaucoma and Neuroophthalmology, Eye Clinic, DINOGMI, University of Genoa, Viale Benedetto XV 5, 16132 Genoa, Italy

## Abstract

*Purpose*. To evaluate if paracentral hyperautofluorescence (HAF) retinal regions, which can be occasionally found and analyzed by optical coherence tomography (OCT), were related to retinal layer changes and to detect which layer was involved. *Methods*. This is a cross-sectional and retrospective study. 648 OCT files were revised. OCTs that showed a paracentral HAF area by using the fundus autofluorescence imaging in Heidelberg Spectralis (Heidelberg Engineering, Germany) were selected. Then retinal layer morphology was analyzed observing OCT scans and a retinal thickness was measured. *Results*. 31 patients were selected: 20 patients had chronic serous epitheliopathy (CSE), 8 patients had resolved central serous chorioretinopathy (CSC), and 3 patients wet age related macular degeneration (ARMD). The HAF zones corresponded to areas of thickness reduction of the external hyporeflective band. In all these areas the retinal pigment epithelium was not atrophic and the neuroepithelium was more or less dystrophic. In particular the retinal thickness was 264 um, 232 um, and 243 um in wet ARMD, CSE, and CSC, respectively; the reduction was significant (*P* < 0.01) compared to the same area of the other eye. *Discussion*. The presence of HAF imaging might be mostly due to a “window effect” rather than an accumulation of lipofuscin.

## 1. Introduction

The predominant fluorophores arising from the fundus have been shown to be located within the retinal pigment epithelium (RPE) lipofuscin (LF) [1], which derives primarily from phagocytosed photoreceptor outer segments. These fluorophores most likely accumulate in RPE cells because the structures of the fluorophores are unusual and not amenable to degradation, rather than because the lysosomal enzymes are defective in these cells [2]. Progressive LF buildup is mainly caused by incomplete degradation of photoreceptor outer segment disks with subsequent incomplete release of degraded material. Over the course of a lifetime, each RPE cell phagocytoses more than 3,000,000,000 outer segments [3–5].

Fundus autofluorescence (AF) imaging is a method that allows topographic mapping of LF distribution in the retinal pigment epithelium cell monolayer as well as of other fluorophores that may occur with disease in the outer retina and the subneurosensory. At the posterior pole autofluorescence is dependent on either outer segment metabolism with an increase of LF concentration [6] or a window effect for the decrease or lack of pigment along plexiform layer which covers the physiological AF [7].

The aim of this study was to evaluate if paracentral hyperautofluorescence (HAF) retinal regions, which can occasionally be found by a user and analyzed by optical coherence tomography (OCT), were related to retinal layer changes and to detect which layer was involved.

## 2. Patient and Methods

This is a retrospective, cross-sectional study. This study followed the principles of the Declaration of Helsinki.

Patients were imaged using the Heidelberg Spectralis HRA + OCT (Heidelberg Engineering, Heidelberg, Germany) in Spectral Domain SD-OCT mode, using a scan field of 30 degrees horizontally and 15 degrees vertically and 19 to 25 OCT horizontal sections (one section at least every 240 *μ*m). The Heidelberg Spectralis HRA + OCT (Heidelberg Engineering, Heidelberg, Germany; Software version 1.6.1.0) can be used in any one of six imaging modes, that is, SD-OCT, fluorescein angiography, indocyanine green angiography, autofluorescence, red-free, and infrared imaging. This paper details use of the instrument in SD-OCT mode only. The Heidelberg Spectralis utilizes a broadband light source centered at 870 nm (i.e., no visible light “beacon”) to simultaneously measure multiple wavelengths, a prerequisite of SD-OCT imaging. Simultaneous confocal scanning laser ophthalmoscopy is used to generate high-resolution images of the retinal surface, thereby providing precise location information of each A-scan within a cross-sectional SD-OCT image. SD-OCT scanning generates 40,000 A-scans/second with an axial resolution of 3.5 microns/pixel digital (7 microns optical) and a transverse resolution of 14 microns [8]. Alignment software continuously tracks any eye movement during image acquisition and then adjusts the position of the A-scan on the retinal surface to ensure accurate registration of cross-sectional OCT images. Using eye tracking and registration technology, multiple images are obtained from a precise location to then be averaged and filtered to remove random noise from the final image. The same eye tracking/registration technology is used to ensure that the instrument automatically rescans images that are influenced by blink artifacts. Similarly, follow-up images are derived from the same area of retina, thereby eliminating subjective placement of the scan by the operator. The SD-OCTs can also scan the fundus with a low-power optically pumped semiconductor laser (*λ* > 488 nm) to elicit autofluorescence, which is detected through a barrier filter (*λ* > 500 nm) and captured at rate of 16 frames per second over a 30° × 30° or a 55° × 55° field (768 × 768 pixels). To enhance the signal-to-noise ratio, 30 to 100 single-line OCT frames were averaged during simultaneous fundus AF and SD-OCT imaging. In each eye, several OCT line scans through separate regions with increased AF were obtained to examine the retinal architecture [9].

### 2.1. Patient's Inclusion/Exclusion Criteria

All the patients were referred to LB for an OCT analysis, and only the OCTs with AF images done from January 2011 to June 2012 were revised. To find patients with a paracentral hyperautofluorescence (HAF) retinal region by OCT, all the AF images were revised. Patients with unilateral disease were considered in the study.

Then all the OCT scans were subjectively analyzed by the three users just observing the Spectralis screen to value if the retinal layers were modified and to distinguish which retinal layers were involved [10, 11].

For HAF areas, the following features of the outer retina were analyzed: the morphological alteration of the EPR, the outer nuclear layer (ONL), and the outer plexiform layer (OPL).

### 2.2. Statistical Analysis

Student's *t*-test was used to compare the thickness of the HFA areas with the corresponding area of the opposite healthy eye. A *P* value >0.05 was considered statistically significant.

## 3. Results

**Figure 1:**
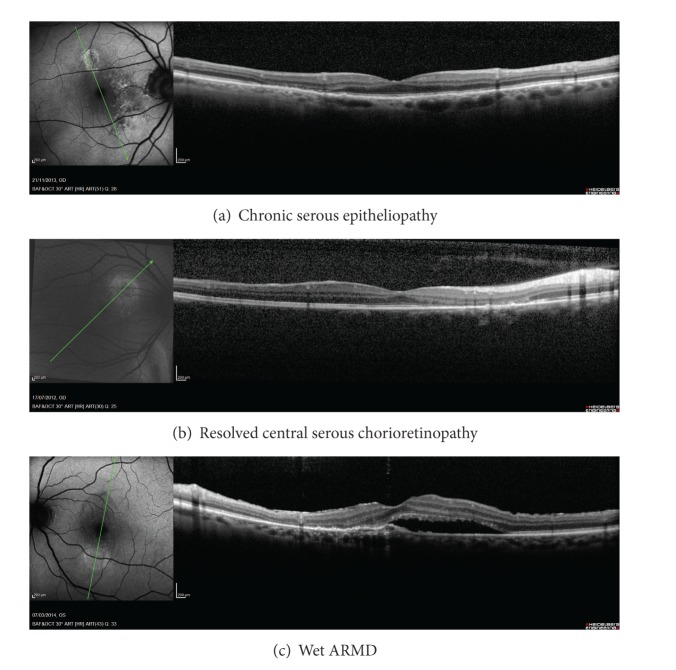
(a) Chronic serous epitheliopathy, (b) resolved central serous chorioretinopathy, and (c) wet ARMD.

Six hundred and 48 patients' files were revised and 31 eyes of 31 patients had a paracentral HAF region. Twenty patients had chronic serous epitheliopathy, eight patients had resolved central serous chorioretinopathy, and three patients had wet ARMD (Table 1) (Figure 1).

**Figure 2:**
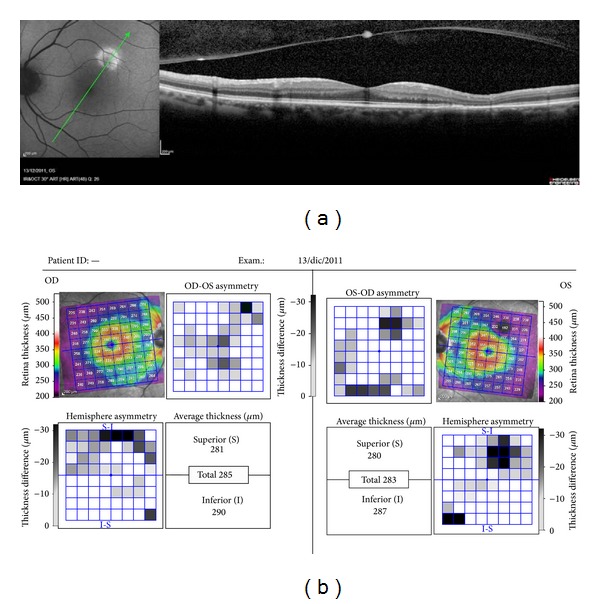
All HAF regions showed a retinal thickness reduction, which when compared to the corresponding area of the other eye showed a significant change.

**Figure 3:**
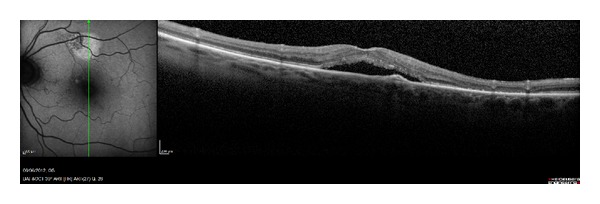
In all the HAF areas the RPE was not atrophic.

**Figure 4:**
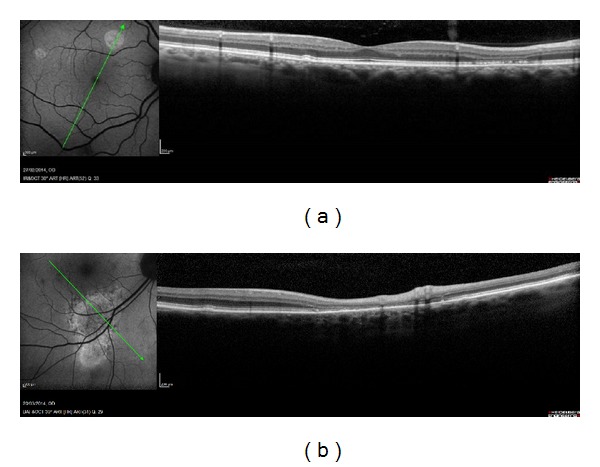
When the outer hyporeflective band was thin, the neuroepithelium was more or less dystrophic.

All HAF regions showed a retinal thickness reduction, which when compared to the corresponding area of the other eye showed a significant change (Table 2, Figure 2). In all these areas the RPE was not atrophic (Figure 3). The HAF intensity was related to the thickness reduction or atrophy of the outer hyporeflectiveband, corresponding to the ONL and to a part of the OPL or Henle Fiber Layer. When this band was thin, the neuroepithelium was more or less dystrophic (Figure 4). In 19 patients (61.29%) the external hyperreflective band was found irregular (Figure 3) (Table 3).

## 4. Discussion

Beyond normal aging processes, LF accumulation is thought to represent a common downstream pathogenetic mechanism in various blinding hereditary and complex retinal diseases, including age related macular degeneration and inherited retinal dystrophies, and Stargardt disease [12–17].

Fundus AF imaging is a clinical tool that allows evaluation of the interaction between photoreceptor cells and RPE in macular disease. The predominant fluorophores arising from the fundus have been shown to be located within the RPE LP [1]. LP is a pigment that exhibits a characteristic AF when excited in ultraviolet or blue light [18].

A decreased AF may indicate photoreceptor death and RPE atrophy or increased RPE melanin content or absorption from extracellular material or cells or fluid which is anterior to RPE. On the other hand, an increased AF might suggest a compromised RPE function related to an ongoing metabolic demand [18–21].

In a normal fundus, the distribution of AF is diffuse with decreased intensity at the optic nerve head, under the retinal blood vessels which appear dark, and at the macula [1, 18]. Macular AF is attenuated by the luteal pigment, and the concentration of this pigment in the fovea is most dense along the outer plexiform layer [22].

Abnormal accumulation of LF produces abnormally increased HAF. Retinal-choroidal diseases, which caused an increased shedding of photoreceptor outer segments, disrupted RPE phagocytic function, or an ability of the RPE to recycle metabolites, produced hyperfluorescence because of LF accumulation as seen in age related macular degeneration and inherited retinal diseases [23].

In 1984 Snodderly et al. showed in primate retinas that most of the pigment in fovea is along the outer plexiform layer, interposed between the foveal photoreceptors and the stimulating light [22]. In our study we found that HAF correlates with thickness reduction in the retinal outer layers and in particular with the thickness reduction or atrophy of the external hyporeflective OCT band which is the ONL and a part of the OPL or Henle fiber layer.

**Figure 5:**
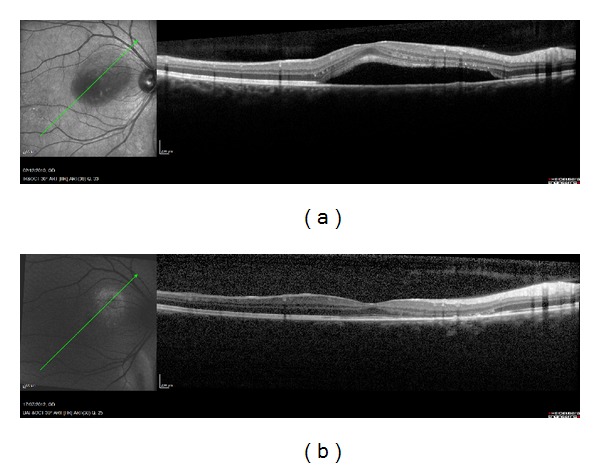
(a) Chronic serous epitheliopathy (CSE) and (b) resolution of the CSE; a retinal thinning with an atrophy of the outer retinal layers (ONL and OPL) can be seen.

These data suggested the possibility that the presence of HAF could be due to a “window effect” for the OPL thinning rather than an accumulation of LP. Clinically, after a localized serous retinal detachment due to different pathologies, it is possible to find hyperautofluorescence areas in that area together with a retinal thinning with an atrophy of the outer retinal layers (ONL and OPL) (Figure 5).

In conclusion our observation suggests that the presence of HAF could be considered the easiest sign to detect retinal thinning and in particular a reduction of ONL and OPL.

## Figures and Tables

**Table 1:** Descriptive analysis of the included patients.

Disease	Age	Retinal thickness
Wet ARMD (*n* = 3)	73.3 (10)	264.7 (54.24)
CSE (*n* = 20)	64.3 (10.5)	232.3 (48.09)
CSC (*n* = 8)	44.3 (6.9)	243.13 (39.68)

*n*: number of eyes, mean (standard deviation), ARMD: age related macular degeneration, CSE: chronic serous epitheliopathy, CSC: resolved central serous chorioretinopathy.

**Table 2:** Comparison of the HFA area with the corresponding area in the healthy eye.

Disease	Involved area (IA)	Corresponding area in the opposite eye (CIA)	Difference (CIA − IA)	*P* value
Wet ARMD	264.7 (54.24)	312.3 (47.12)	47.67 (8.74)	*P* < 0.01
CSE	232.3 (48.09)	306.65 (38.96)	71.85 (28.88)	*P* < 0.001
CSC	243.13 (39.68)	307 (47.02)	63.88 (27.67)	*P* < 0.001

Mean (standard deviation), IA: the area involved by the pathology, CIA: the corresponding IA in the contralateral eye, ARMD: age related macular degeneration, CSE: chronic serous epitheliopathy, CSC: resolved central serous chorioretinopathy.

**Table 3:** Qualitative changes of retinal layers.

Morphological change of	Dystrophic neuroepithelium	Retinal thickness reduction	Hyperautofluorescence
Wet ARMD (*n* = 3)	3++	3	3

CSE (*n* = 20)	8+	20	13
	12++		

CSC (*n* = 8)	1+	8	3
	7−		

ARMD: age related macular degeneration, CSE: chronic serous epitheliopathy, CSC: resolved central serous chorioretinopathy, *n* = number of eyes.

Dystrophic neuroepithelium was assessed from less “−” to more “+” or “++.”

## References

[1] Delori FC, Dorey CK, Staurenghi G, Arend O, Goger DG, Weiter JJ (1995). In vivo fluorescence of the ocular fundus exhibits retinal pigment epithelium lipofuscin characteristics. *Investigative Ophthalmology and Visual Science*.

[2] Cuervo AM, Dice JF (2000). When lysosomes get old. *Experimental Gerontology*.

[3] Wing GL, Blanchard GC, Weiter JJ (1978). The topography and age relationship of lipofuscin concentration in the retinal pigment epithelium. *Investigative Ophthalmology and Visual Science*.

[4] Feeney-Burns L, Berman ER, Rothmann H (1980). Lipofuscin of human retinal pigment epithelium. *American Journal of Ophthalmology*.

[5] Weiter JJ, Delori FC, Wing GL, Fitch KA (1986). Retinal pigment epithelial lipofuscin and melanin and choroidal melanin in human eyes. *Investigative Ophthalmology and Visual Science*.

[6] Schmitz-Valckenberg S, Holz FG, Bird AC, Spaide RF (2008). Fundus autofluorescence imaging: review and perspectives. *Retina*.

[7] Bottoni F, Carmassi L, Cigada M, Moschini S, Bergamini F (2008). Diagnosis of macular pseudoholes and lamellar macular holes: is optical coherence tomography the “gold standard”?. *The British Journal of Ophthalmology*.

[8] Heidelberg Enginneering Spectralis hardware operating instructions.

[9] Chen FK, Patel PJ, Coffey PJ, Tufail A, da Cruz L (2010). Increased fundus autofluorescence associated with outer segment shortening in macular translocation model of neovascular age-related macular degeneration. *Investigative Ophthalmology and Visual Science*.

[10] Tanner V, Chauhan DS, Jackson TL, Williamson TH (2001). Optical coherence tomography of the vitreoretinal interface in macular hole formation. *British Journal of Ophthalmology*.

[11] Haouchine B, Massin P, Tadayoni R, Erginay A, Gaudric A (2004). Diagnosis of macular pseudoholes and lamellar macular holes by optical coherence tomography. *The American Journal of Ophthalmology*.

[12] Eagle RC, Lucier AC, Bernardino VB, Yanoff M (1980). Retinal pigment epithelial abnormalities in fundus flavimaculatus: a light and electron microscopic study. *Ophthalmology*.

[13] Miller SA (1978). Fluorescence in Best's vitelliform dystrophy, lipofuscin, and fundus flavimaculatus. *British Journal of Ophthalmology*.

[14] O'Gorman S, Flaherty WA, Fishman GA, Berson EL (1988). Histopathologic findings in Best's vitelliform macular dystrophy. *Archives of Ophthalmology*.

[15] Dorey CK, Wu G, Ebenstein D, Garsd A, Weiter JJ (1989). Cell loss in the aging retina. Relationship to lipofuscin accumulation and macular degeneration. *Investigative Ophthalmology and Visual Science*.

[16] Steinmetz RL, Garner A, Maguire JI, Bird AC (1991). Histopathology of incipient fundus flavimaculatus. *Ophthalmology*.

[17] Katz ML (2002). Potential role of retinal pigment epithelial lipofuscin accumulation in age-related macular degeneration. *Archives of Gerontology and Geriatrics*.

[18] Delori F, Keilhauer C, Sparrow JR, Staurenghi G, Holz FG, Schmitz-Valckenberg S, Spaide RF, Bird AC (2007). Origin of fundus autofluorescence. *Atlas of Fundus Autofluorescence Imaging*.

[19] Kennedy CJ, Rakoczy PE, Constable IJ (1995). Lipofuscin of the retinal pigment epithelium: a review. *Eye*.

[20] Lima LH, Greenberg JP, Greenstein VC (2012). Hyperautofluorescent ring in autoimmune retinopathy. *Retina*.

[21] Von Ruckmann A, Fitzke FW, Bird AC (1995). Distribution of fundus autofluorescence with a scanning laser ophthalmoscope. *British Journal of Ophthalmology*.

[22] Snodderly DM, Auran JD, Delori FC (1984). The macular pigment. II. Spatial distribution in primate retinas. *Investigative Ophthalmology and Visual Science*.

[23] Holz FG, Schmitz-Valckenberg S, Spaide RF, Bird AC (2007). *Atlas of Fundus Autofluorescence Imaging*.

